# Altered network homogeneity of the default mode network in patients with tension-type headache

**DOI:** 10.3389/fneur.2026.1870552

**Published:** 2026-09-01

**Authors:** Huan Yin, Xiaohong Guo, Yujun Gao, Qingzhen Zhou, Peng Min, Min Xia, Xiao Peng, Ling Chen, Yi Wang, Qi Han

**Affiliations:** 1 Department of Neurology, Sinopharm Dongfeng General Hospital, Hubei University of Medicine, Shiyan, Hubei, China; 2 Department of Psychiatry, Tianyou Hospital Affiliated to Wuhan University of Science and Technology, Wuhan University of Science and Technology, Wuhan, Hubei, China; 3 Department of Medical Imaging, Sinopharm Dongfeng General Hospital, Hubei University of Medicine, Shiyan, Hubei, China

**Keywords:** default mode network, network homogeneity, resting-state functional magnetic resonance, support vector machine, tension-type headache

## Abstract

**Objective:**

Dysfunction of the default mode network (DMN) is strongly linked to pain processing, as well as onset and progression of chronic pain disorders; however, its specific role in the pathogenesis of tension-type headache (TTH) remains incompletely understood. This study aims to explore the network homogeneity (NH) of the DMN in the pathophysiological mechanisms underlying TTH.

**Methods:**

Resting-state functional magnetic resonance imaging (rs-fMRI) and clinical headache scale assessments were conducted on 68 patients with TTH and 63 healthy controls (HCs). Independent component analysis and NH analysis were used to evaluate the neuroimaging data.

**Results:**

Patients with TTH exhibited decreased NH values in the right posterior cingulate cortex (PCC) and increased NH values in the right medial superior frontal gyrus (SFGmed), bilateral angular gyrus (AG), and left precuneus (PCu), compared to HCs. Additionally, the NH values of right AG, left AG and right PCC were positively correlated with Headache Impact Test-6 (HIT-6), Migraine Disability Assessment Test (MIDAS) scores and headache intensity. Furthermore, support vector machine (SVM) analysis revealed that NH values in the right PCC could distinguish TTH patients from HCs with an accuracy of 75.57%, sensitivity of 84.13%, specificity of 70.59%, and an AUC of 0.7156.

**Conclusion:**

This study demonstrates altered network homogeneity (NH) within the DMN in patients with TTH. Furthermore, the NH value of the right PCC showed discriminative potential to distinguish TTH patients from healthy controls via SVM classification.

## 1 Introduction

Tension-type headache (TTH) is the most prevalent primary headache disorder globally. Its clinical manifestations include mild to moderate, bilateral, non-pulsating headache, typically without associated symptoms such as nausea, vomiting, or sensitivity to light and sound (1). According to data from the 2021 Global Burden of Disease Study, the global prevalence of TTH has reached 2.01 billion cases. Its chronic and protracted course not only leads to significant functional disability among patients but also imposes a heavy socioeconomic burden (2). However, the diagnosis of TTH currently relies primarily on clinical history, and its underlying neurobiological mechanisms have not yet been fully elucidated, which significantly limits our understanding of the pathophysiological processes underlying this condition.

In recent years, resting-state functional magnetic resonance imaging (rs-fMRI) is widely used in studies of brain function owing to its non-invasive nature and excellent spatial resolution. While standard 3 T rs-fMRI lacks high temporal resolution, its sampling performance is adequate for capturing low-frequency spontaneous BOLD fluctuations intrinsic to large-scale brain networks. Previous studies have demonstrated that patients with TTH exhibit significant abnormalities in both static and dynamic functional connectivity within the default mode network (DMN) (3). The DMN, as a highly active functional neural network system in the brain at rest (4, 5), is primarily composed of the medial prefrontal cortex (mPFC), posterior cingulate cortex (PCC), precuneus (PCu), angular gyrus (AG), the temporoparietal junction (TPJ), and the parahippocampal gyrus (6, 7). Its core functions encompass self-referential cognition, emotional regulation, memory retrieval, and the integration of pain perception. The structural and functional integrity of this network is crucial for maintaining normal brain homeostasis (8–10). Current research indicates that abnormalities in DMN functional connectivity have been observed in patients with various pain conditions (11–15), suggesting that pain has a broad impact on the DMN. In addition, the DMN is involved in pain inhibition and influences the efficiency of pain processing (16, 17). These studies suggest that the DMN plays a key role in the pathophysiological mechanisms of TTH. Therefore, a thorough elucidation of the functional characteristics of the DMN in patients with TTH is expected to provide an important theoretical basis for uncovering the underlying pathophysiological mechanisms of TTH.

Currently, research on DMN functional abnormalities in TTH patients remains limited. Existing studies primarily focus on significant static and dynamic functional connectivity abnormalities in the DMN of TTH patients (3), yet they still have notable limitations: First, most existing studies focus on point-to-point connectivity changes within specific brain regions of the DMN, lacking a systematic assessment of the network’s overall functional coherence; Second, traditional functional connectivity analyses largely rely on predefined regions of interest (ROIs), which can only reflect the relative strength of local connections. This approach struggles to capture holistic and synergistic disruptions at the network level and is susceptible to the subjectivity of ROI definitions and individual anatomical variations, thereby limiting the objectivity and comprehensiveness of research findings. It is worth noting that network homogeneity (NH), as a novel voxel-level brain network analysis method, directly reflects the overall functional coherence and consistency of a network by quantifying the mean of the time-series correlations between an individual voxel and all other voxels within its network (18). Compared to traditional analytical methods, NH offers significant advantages: (1) It does not require predefined ROIs, enabling unbiased analysis at the level of all network voxels. This effectively avoids biases resulting from subjective selection and captures the functional integration characteristics of the entire network more comprehensively; (2) By focusing on the degree of coordination among all voxels within the network rather than local point-to-point connections, it more accurately reflects the overall efficacy of the network as a functional unit, making it particularly suitable for revealing cross-regional coordination disorders; (3) It exhibits higher sensitivity to weak but widespread functional connectivity abnormalities within the network, effectively addressing the shortcomings of traditional methods that focus solely on significant local connections and are prone to overlooking diffuse network abnormalities (19, 20). This method has been successfully applied to brain network studies of conditions such as mania (18), attention-deficit/hyperactivity disorder (19), epilepsy (20), and depression (21). However, there is currently a lack of systematic research on the homogeneity of the DMN network in TTH patients, and this research gap significantly limits in-depth exploration of the pathological mechanisms underlying TTH brain networks. At the same time, the clinical diagnosis of TTH primarily relies on the symptomatic criteria established by the International Headache Society (IHS) (22). The lack of objective, quantifiable neuroimaging markers significantly impairs the accuracy and standardization of diagnosis and treatment; however, abnormalities in brain networks offer a potential technical approach to addressing this clinical challenge. Support vector machine (SVM), as a pattern recognition algorithm based on statistical learning theory, offers high generalization capabilities and the advantage of learning from small datasets and can be used to accurately distinguish diseases by analyzing abnormal features in brain networks (23). Currently, this technology has been widely applied in imaging classification studies of neurological disorders such as schizophrenia (23), migraine (24), epilepsy (25), and major depressive disorder (26), confirming its value in the objective diagnosis of these conditions.

Based on the above research background and existing gaps, this study proposes the following scientific hypotheses: (1) Compared with healthy controls (HCs), TTH patients exhibit altered NH in the DMN, which may reflect the disordered state of functional integration within the network; (2) The NH changes in specific brain regions within the DMN are correlated with clinical indicators reflecting TTH severity and functional impact; (3) Based on the NH characteristics of the DMN, SVM can effectively distinguish TTH patients from HCs. This study employs resting-state fMRI to systematically characterize DMN functional homogeneity via NH analysis and SVM classification. Notably, our design only includes healthy volunteers as the control group, without additional cohorts suffering from other chronic pain. Therefore, the DMN NH abnormalities identified herein cannot be confirmed as pathognomonic signatures unique to TTH. This work still helps elucidate central neural mechanisms of TTH, provides objective neuroimaging references for clinical assessment, and lays experimental groundwork for subsequent research exploring precise diagnosis and targeted treatments for headache disorders.

## 2 Materials and methods

### 2.1 Subjects

This study included 68 TTH patients and 63 HCs who were matched for age, sex, and educational level, all of whom were treated at the Department of Neurology at Shiyan Sinopharm Dongfeng General Hospital between January 2024 and April 2025. This study was approved by the Ethics Committee of Sinopharm Dongfeng General Hospital (Approval No.: LW-2024-064), which is affiliated with Hubei Medical College. All participants signed written informed consent forms. The diagnosis of TTH was strictly based on the criteria of the International Classification of Headache Disorders, 3rd Edition (ICHD-3), and was independently confirmed by two neurologists. The inclusion criteria for patients were as follows: (1) age 18 to 60 years; (2) right-handed; (3) in a headache-free period at the time of scanning: no headache episodes during the 3 days prior to the scan, on the day of the scan, or the day following the scan; (4) no use of preventive medication within at least 14 days prior to the MRI examination; (5) no history of neurological or psychiatric disorders; (6) no contraindications for MRI. The exclusion criteria were as follows: (1) history of other types of headaches or chronic pain conditions; (2) history of alcohol, nicotine, or drug abuse; (3) intracranial lesions; (4) history of severe neurological disorders or psychiatric conditions; (5) pregnancy or lactation. The inclusion criteria for HCs were as follows: (1) age 18 to 60 years; (2) right-handed; (3) no history of neurological or psychiatric disorders; (4) no contraindications for MRI. The exclusion criteria were identical to those applied to TTH patients.

### 2.2 Scale evaluation

TTH patients were evaluated by trained physicians using the following validated scales: the 6-item Headache Impact Test (HIT-6) (27) was used to assess the overall impact of headache on daily functions; the Migraine Disability Assessment Test (MIDAS) (28) was used to assess the number of days of functional disability caused by headache in the past 3 months. This scale has been widely applied in TTH research.

### 2.3 MRI acquisition

MRI data were acquired using a Philips Elition S 3.0 T scanner equipped with a 32-channel head coil. During scanning, participants were instructed to lie supine, remain relaxed, keep their eyes closed, and avoid engaging in specific thoughts. Earplugs were used to mitigate the effects of MRI noise, while foam pads were employed to limit head movements. Assessments were conducted by a senior MRI technologist. The rs-fMRI scanning parameters were as follows: field of view (FoV) = 220 × 220 mm, matrix size = 64 × 64, flip angle (FA) = 90°, repetition time (TR) = 2000 ms, echo time (TE) = 30 ms, and slice thickness = 5 mm. In addition, three-dimensional T1-weighted images were generated as follows: FoV = 240 × 240 mm, matrix size = 256 × 256, FA = 90°, TR = 8.35 ms, TE = 3.27 ms, and slice thickness = 1 mm.

### 2.4 Data preprocessing

Data preprocessing was performed using the Dynamic Parametric Analysis of Resting-State Functional MRI (DPARSF) tool in MATLAB 2013b (29). In the first stage of preprocessing, scan data from the first 10 time points were discarded to eliminate initial signal instability; subsequently, slice timing correction and head motion correction were performed sequentially to optimize the quality of the image time series. Strict thresholds were set for head motion screening: if the maximum displacement of the subject in the X, Y, and Z axes was > 2 mm or the maximum rotation angle was >2° (30), the scan was repeated on the same day until the motion criteria were met.

After registering individual structural images with corresponding functional images, image tissue segmentation was performed to separate gray matter, white matter, and cerebrospinal fluid tissues. Using the parameters obtained from co-registration, individual echo planar imaging (EPI) images were normalized to the Montreal Neurological Institute (MNI) standard space, and the voxel size was resampled to 3 × 3 × 3 mm^3^. During the functional image normalization stage, head motion parameters, white matter signals, and cerebrospinal fluid signals were introduced as regressors to effectively eliminate interference signals.

Subsequent preprocessing steps were performed sequentially: band-pass filtering of the images in the 0.01 ~ 0.08 Hz frequency band, spatial smoothing with a 6 mm full width at half maximum (FWHM) Gaussian kernel, and linear trend removal to reduce the interference of low-frequency drift and physiological high-frequency noise on the data. Global signal regression was not performed during preprocessing in this study; instead, the global signal was retained to avoid artifact introduction and distortion of resting-state functional connectivity patterns. This processing method is particularly critical for clinical research populations, as global signal regression can easily exert significant interference on research results (31).

### 2.5 DMN identification

Spatial independent component analysis (ICA) was used in this study to identify the core components of the DMN in TTH patients and HCs through the group ICA tool of the GIFT-fMRI toolbox, with the process including three steps: data dimensionality reduction, independent component separation, and inverse reconstruction (32).

First, principal component analysis (PCA) was used to perform spatial dimensionality reduction on the group fMRI data, and the dimensionality-reduced data of all subjects were integrated; then, the minimum description length (MDL) criterion was used to automatically determine the optimal number of independent components (ICs), and the ICASSO algorithm was used to cluster multiple ICA results to ensure the reliability of group-level ICs. Based on the dimensionality reduction of group-level ICs and the inverse reconstruction of PCA results, subject-specific ICs were obtained; combined with the GIFT DMN standard template and visual inspection, group-level components significantly connected to the core brain regions of the DMN were retained, and one core DMN component was identified for all subjects.

Group-level DMN component significance testing was performed using REST software: statistical maps were generated, with a voxel-based one-sample *t*-test (*p <* 0.001) as the initial threshold, and multiple comparisons were corrected by Gaussian random field (GRF) theory (both cluster and voxel levels were *p <* 0.01). Individualized masks were constructed for the verified DMN components and merged into a comprehensive DMN mask (voxel value set to 1), which covered the core DMN brain regions such as PCC, precuneus, mPFC, bilateral TPJ, and parahippocampal gyrus, ensuring the inclusion of core nodes and connection pathways of the network.

### 2.6 NH analysis

NH analysis was used to measure the consistency of neural activity between each voxel and its adjacent voxels. In this study, whole-brain NH values were calculated based on the MATLAB 2013b platform (18) and extracted within the DMN mask. For each single voxel, time-series correlation coefficients were computed against all remaining voxels within the predefined DMN mask. Z-transformation was subsequently applied to standardize these correlation coefficients into normalized z-scores for further analyses. The standardized z-images were generated as individual NH maps and subjected to intergroup statistical comparisons. In all between-group analyses of NH metrics, demographic factors including age, sex and educational years were controlled as nuisance covariates. In addition, framewise displacement (FD) of every subject was incorporated as an extra covariate, as subtle head motion across volumes could introduce confounding biases to resting-state fMRI signals.

## 3 Statistical analyses

To compare the demographic and clinical characteristics between TTH patients and HCs, the independent samples *t*-test was adopted to analyze intergroup differences in age, years of education, mean frame-wise displacement (FD), HIT-6 score, MIDAS score, headache intensity, disease duration, and attack frequency, and the χ^2^ test was used to evaluate gender distribution. The significance level was set at *p* < 0.05.

Inter-group comparison of voxel-based DMN NH maps between the TTH patients and HCs was performed using analysis of covariance (ANCOVA), with age, gender, years of education, and FD as covariates to control for the influence of confounding factors. Voxel-level statistical analyses were performed with a primary threshold of *p* < 0.001. Gaussian random field (GRF) cluster-level correction was subsequently applied, with the final statistical threshold set at *p* < 0.01 for identifying significant intergroup brain differences using the REST toolkit.

### 3.1 Correlation analysis

If significant inter-group differences in NH values were detected in DMN brain regions between the TTH patients and HCs, the brain regions with differences were defined as ROIs, and the NH values (after z-standardization) of each ROI were extracted to analyze their correlation with the clinical characteristics of TTH patients (HIT-6 score, MIDAS score, headache intensity, disease duration, and attack frequency). The Benjamini-Hochberg correction method was employed to control for multiple comparisons, with a significance threshold of *p* < 0.05.

### 3.2 SVM analysis

Support vector machine analysis was performed using MATLAB software and the LIBSVM toolbox, which was mainly used to evaluate the ability of NH values in DMN-related brain regions to distinguish TTH patients from healthy controls. The specific process was as follows: first, the extracted NH values of DMN-related brain regions were used as feature variables, and the training set and test set were divided; a classification model was constructed using the LIBSVM tool, and the grid search method was used to determine the kernel function (the RBF kernel function was selected) and optimal parameters (C value, *γ* value); k-fold cross-validation was used to reduce overfitting and improve model stability. Subsequently, leave-one-out cross-validation was used to evaluate the classification effect of the model, and the classification accuracy, sensitivity, and specificity were finally calculated to verify the feasibility of using NH values to distinguish TTH patients from healthy controls (33).

## 4 Results

### 4.1 Demographic and clinical characteristics

**Figure 1:**
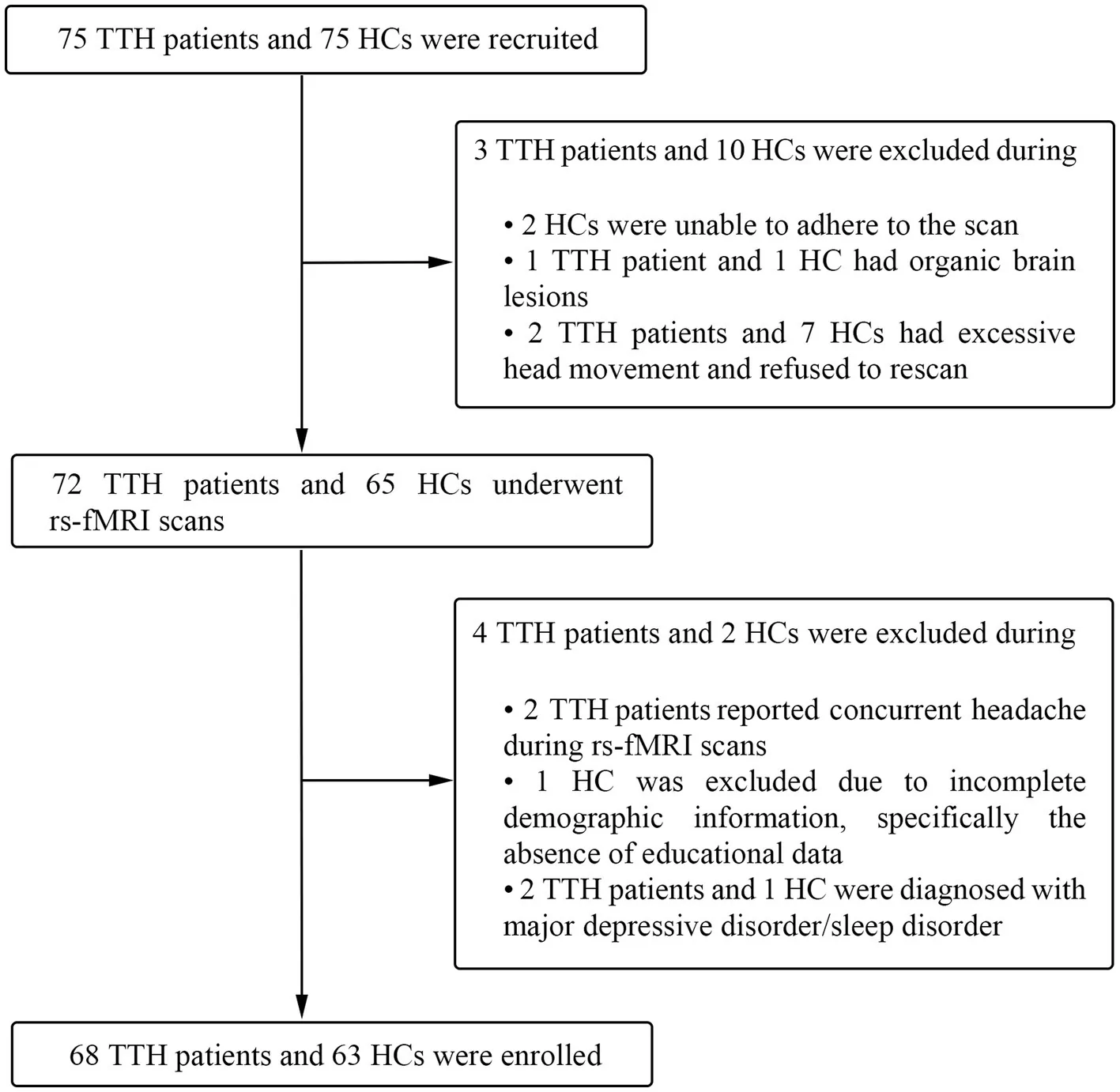
Flow chart of the selection process for all participants. TTH = tension-type headache; HCs = healthy controls; rs-fMRI = resting-state functional magnetic resonance imaging.

A total of 75 TTH patients and 75 HCs were recruited in this study. Two HCs were excluded owing to their inability to undergo an rs-fMRI scan; one TTH patient and one HC were excluded because they were presented with organic intracranial disease; two TTH patients and 7 HCs were excluded because they had excessive head movements and refused a rescan. Furthermore, two TTH patients were excluded since they reported concurrent headaches during the MRI scanning; one HC was excluded due to insufficient demographic information, particularly a lack of educational data; and two TTH patients and one HC were excluded due to a diagnosis with major depressive disorder/sleep disorder. Finally, 68 TTH patients—consisting of 30 males and 38 females—and 63 HCs (comprising 30 males and 33 females) were enrolled in this study (Figure 1). The demographic and clinical characteristics of the two groups are shown in Table 1. There were no significant differences between the two groups in terms of age (*p* = 0.058), gender (*p* = 0.691), and education level (*p* = 0.112).

**Table 1:** Demographic and clinical characteristics of the participants.

Information	TTH	HCs	*p*-value
Gender (M/F)	68(30/38)	63(30/33)	0.691
Age (years)	41.85 ± 6.53	39.51 ± 7.49	0.058
Education (years)	10.47 ± 3.78	11.43 ± 2.99	0.112
HIT-6	55.27 ± 12.93		
MIDAS	15.37 ± 4.27		
Headache intensity (0–10)	5.67 ± 2.06		
Disease duration (years)	1.72 ± 1.39		
Headache frequency (days/month)	5.98 ± 4.77		

TTH = Tension-type headache; HCs = Healthy controls; HIT-6 = Headache Impact Test-6; MIDAS = Migraine Disability Assessment Test.

### 4.2 NH differences in the DMN between groups

**Figure 2:**
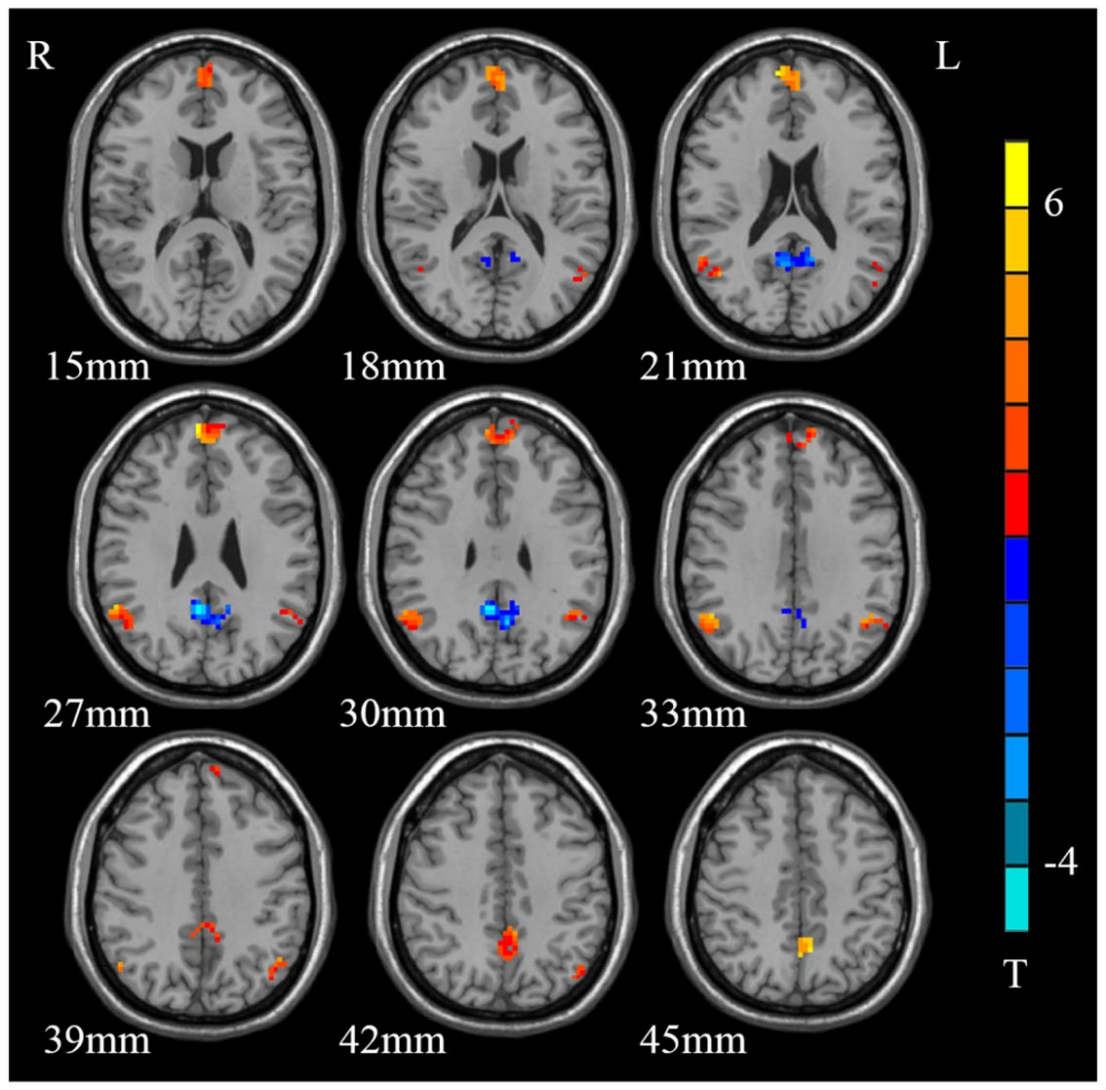
Differences in NH values between TTH and HCs. The color bar represents the t-values for the group analysis. Increased NH values are presented in yellow. Decreased NH values are presented in blue. NH = Network homogeneity; TTH = Tension-type headache; HCs = Healthy controls; R = Right; L = Left.

ANCOVA showed significant inter-group differences in NH values within the DMN between TTH patients and HCs. Compared with HCs, TTH patients exhibited significantly increased NH values in the right medial superior frontal gyrus (SFGmed), left AG, right AG, and left PCu, while the NH value in the right PCC was significantly decreased (see Table 2 and Figure 2).

**Table 2:** Significant differences in NH values between patients with TTH and HCs.

Cluster location	Peak (MNI)			Number of voxels	*T*-value
	X	Y	Z		
Right SFGmed	9	63	24	178	5.79
Right AG	57	−51	24	82	5.07
Left AG	−48	−54	33	53	4.56
Right PCC	6	−51	30	111	−3.96
Left PCu	−3	−54	48	45	5.56

NH = Network homogeneity; TTH = Tension-type headache; HCs = Healthy controls; MNI = Montreal Neurological Institute; SFGmed = medial of superior frontal gyrus; AG = angular gyrus; PCC = posterior cingulate cortex; PCu = precuneus.

### 4.3 Correlation between NH values and HIT-6, MIDAS scores, headache intensity, disease duration, and attack frequency in TTH patients

**Figure 3:**
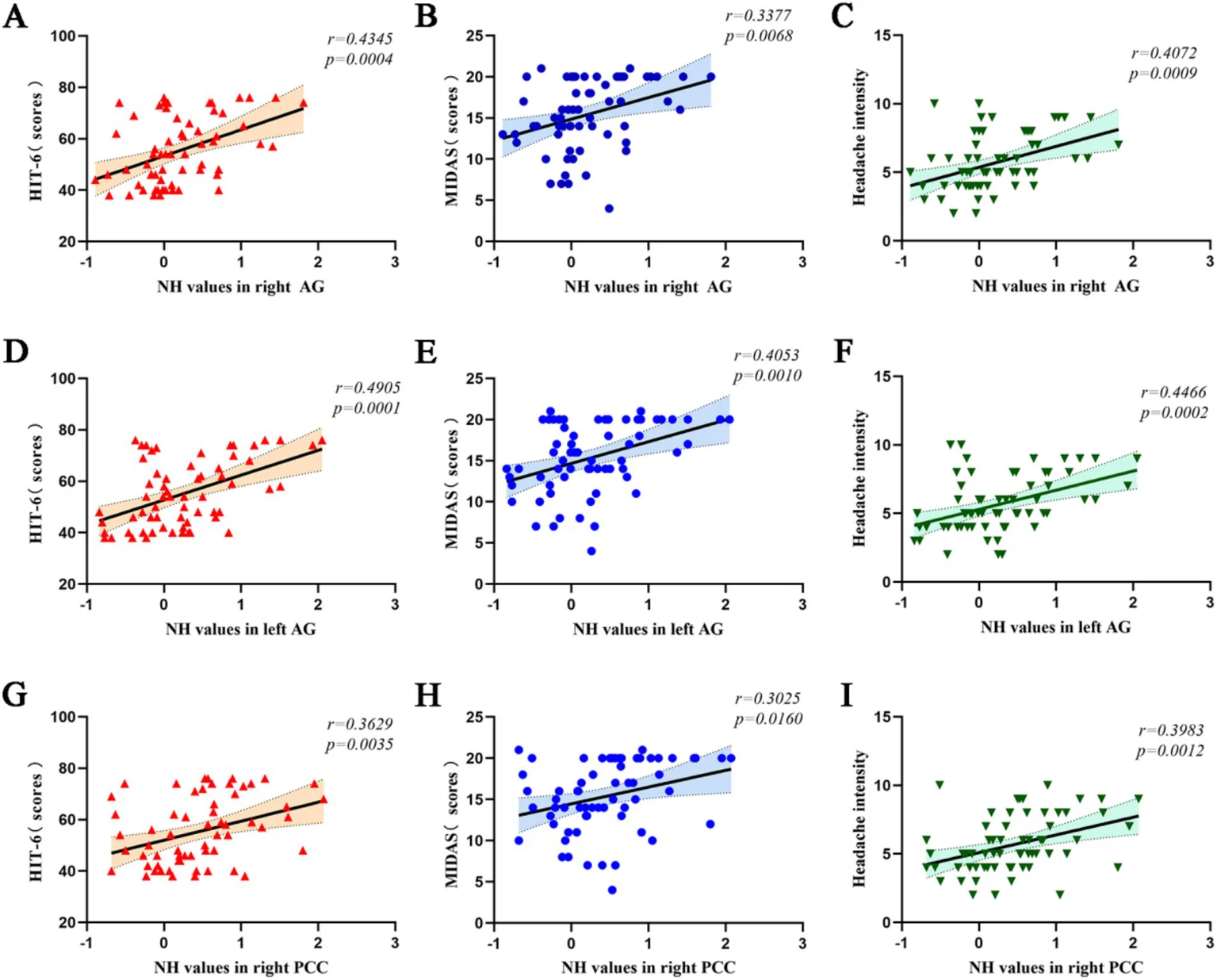
Correlation of NH values with clinical variables in patients with TTH. **(A)** NH value in the right AG showed a positive linear correlation with HIT-6 scores (*r* = 0.4345, raw *p* = 0.0004). **(B)** NH value in the right AG showed a positive linear correlation with MIDAS scores (*r* = 0.3377, raw *p* = 0.0068). **(C)** NH value in the right AG showed a positive linear correlation with headache intensity (*r* = 0.40722, raw *p* = 0.0009). **(D)** NH value in the left AG showed a positive linear correlation with HIT-6 scores (*r* = 0.4905, raw *p* = 0.0001). **(E)** NH value in the left AG showed a positive linear correlation with MIDAS scores (*r* = 0.4053, raw *p* = 0.001). **(F)** NH value in the left AG showed a positive linear correlation with headache intensity (*r* = 0.4466, raw *p* = 0.0002). **(G)** NH value in the right PCC showed a positive linear correlation with HIT-6 scores (*r* = 0.3629, raw *p* = 0.0035). **(H)** NH value in the right PCC showed a positive linear correlation with MIDAS scores (*r* = 0.3025, raw *p* = 0.016). **(I)** NH value in the right PCC showed a positive linear correlation with headache intensity (*r* = 0.3983, raw *p* = 0.0012). NH = network homogeneity; AG = angular gyrus; PCC = posterior cingulate cortex; HIT-6 = Headache Impact Test-6; MIDAS = Migraine Disability Assessment Test.

The mean NH values (z-standardized) of the above 5 differential brain regions were extracted, and Pearson correlation analysis was performed within the TTH patients. After Benjamini–Hochberg FDR correction, the NH values of right AG, left AG and right PCC were positively correlated with HIT-6, MIDAS and headache intensity (see Figure 3); no significant correlation was found between the NH values of the other brain regions and the clinical scores.

### 4.4 SVM and ROC results

**Figure 4:**
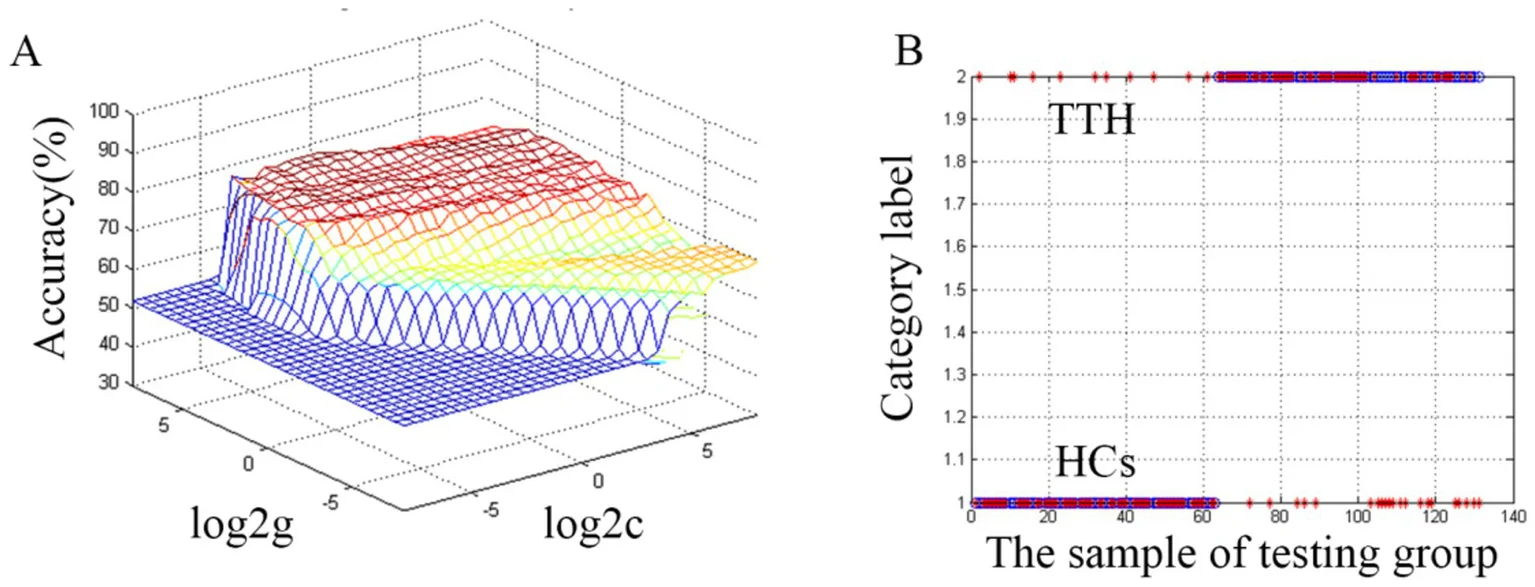
Visualization of SVM classification performed using NH values in the right PCC to differentiate between TTH patients and HCs. **(A)** 3D Visualization of the PCC with the most optimal parameters; **(B)** Classification map of the NH values in the right PCC. SVM = support vector machine; NH = network homogeneity; TTH = tension-type headache; HCs = healthy controls; PCC = posterior cingulate cortex.

**Figure 5:**
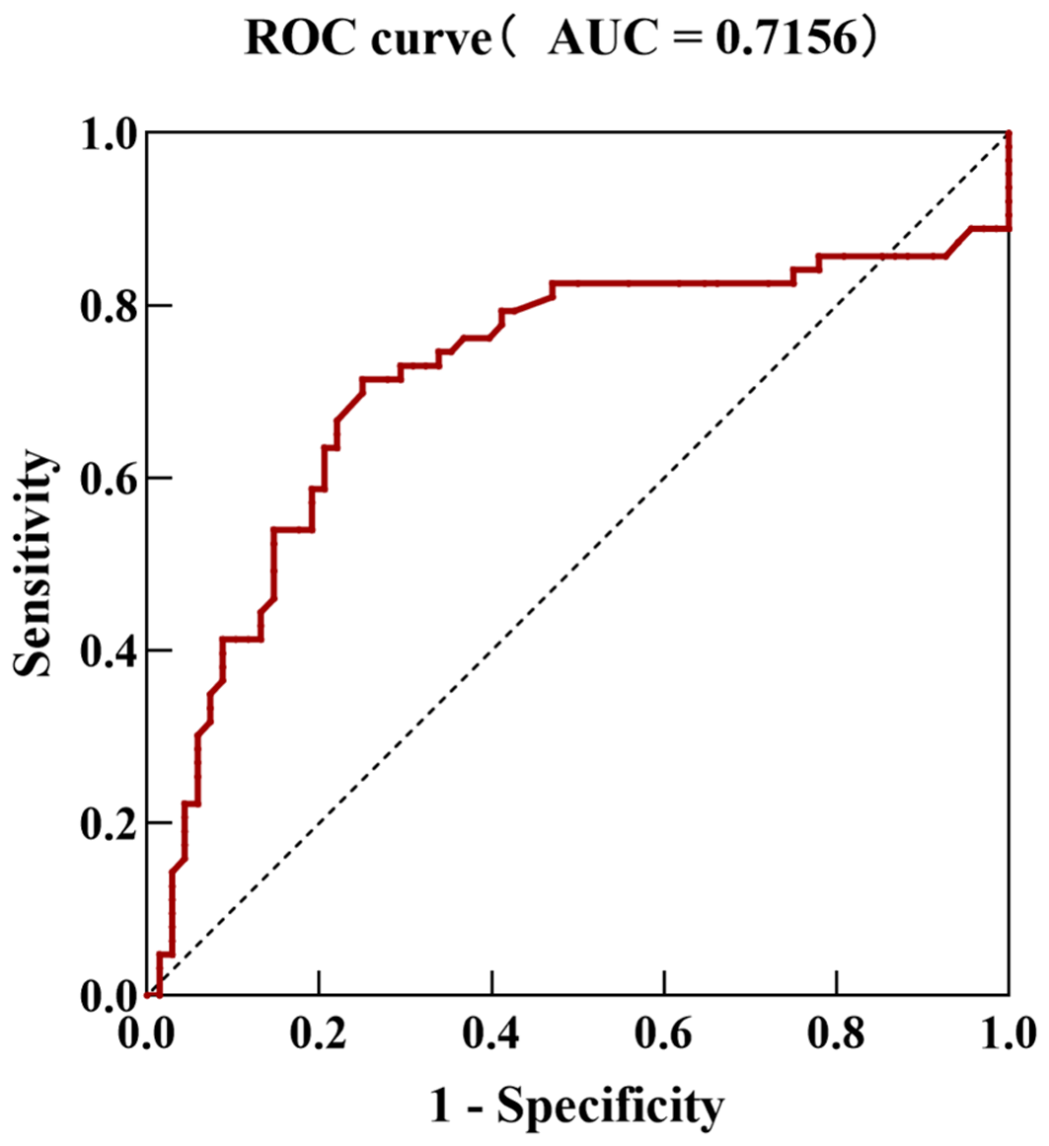
Receiver operating characteristic (ROC) curve of the SVM classifier based on the NH value of the right PCC for distinguishing TTH patients from HCs (AUC = 0.7156). NH = Network Homogeneity; PCC = Posterior Cingulate Cortex.

Support vector machine analysis showed that the NH value of the right PCC exhibited modest discriminative potential to distinguish TTH patients from HCs, with a classification accuracy of 75.57%, sensitivity of 84.13%, and specificity of 70.59% (see Figure 4). The corresponding receiver operating characteristic (ROC) curve analysis yielded an AUC value of 0.7156 (see Figure 5), indicating moderate classification performance of the single-feature model.

## 5 Discussion

Based on rs-fMRI data, this study extracted the DMN using ICA and further explored the functional integration characteristics of the DMN in TTH patients through NH analysis. The results showed that compared with HCs, TTH patients had significant abnormalities in NH values of specific brain regions within the DMN: the NH values in the right SFGmed, bilateral AG, and left PCu were significantly increased, while the NH value in the right PCC was decreased. Correlation analyses demonstrated that the NH values of right AG, left AG and right PCC were positively correlated with HIT-6, MIDAS scores and headache intensity among TTH patients. SVM analysis revealed that right PCC NH showed modest discriminative potential to differentiate TTH patients from healthy controls, with an accuracy of 75.57%, sensitivity of 84.13%, specificity of 70.59%, and an AUC of 0.7156. Collectively, these exploratory findings highlight aberrant DMN functional homogeneity in TTH and provide preliminary evidence for the right PCC NH as a candidate imaging biomarker for TTH pathophysiology.

The SFGmed, as a core subregion of the mPFC, is highly overlapping with the mPFC in anatomical localization and functional properties, and both are key nodes of the DMN (34, 35). Its core functions include emotion regulation, cognitive control, and pain-related attention allocation, and it also plays an indispensable role in self-referential processing and emotional integration (36, 37). In this study, NH analysis found that the NH value of the right SFGmed in TTH patients was significantly higher than that in HCs, and this result was directly supported by relevant studies in the field of TTH. Zhang et al. (38) clearly confirmed in a study involving 33 TTH patients that the regional homogeneity (ReHo) value of the right SFGmed in patients was significantly increased in the conventional frequency band (0.01–0.08 Hz), and this functional abnormality was more significant in the pain-related slow 5 frequency band (0.01–0.027 Hz) with a wider spatial distribution. Meanwhile, Wang et al. (39) found that in the slow wave 5 frequency band (0.01–0.027 Hz), compared with HCs, the functional connectivity (FC) between the right SFGmed and the right medial temporal pole/right inferior temporal gyrus was significantly enhanced in TTH patients. The increased NH value of SFGmed observed in this study is highly consistent with the abnormal ReHo value reported by Zhang et al. (38) and the abnormal FC value reported by Wang et al. (39), collectively confirming that hyperfunction of SFGmed is a characteristic brain function change in TTH patients, and its core mechanism is the abnormal enhancement of local neural activity synchronization in this brain region. It is worth noting that this functional abnormality is not unique to TTH and has also been verified in migraine studies. Coppola et al. (40) used rs-fMRI seed point analysis in patients with migraine without aura during acute attacks and found that the FC between mPFC and PCC, as well as bilateral insula, within the DMN was significantly enhanced in migraine patients. Essentially, it reflects the common neural mechanism of abnormal functional integration in the midline core region of the DMN in patients with primary headaches (including TTH and migraine), providing important neuroimaging evidence for the hypothesis that different types of primary headaches may share similar central sensitization pathways.

The AG, serving as a cross hub between the DMN, the sensorimotor network, and the attentional network, mainly mediates multimodal sensory integration, memory retrieval, and the processing of pain-related information, and plays an indispensable role in the integrated regulation of somatosensory and cognitive signals (41). In this study, elevated NH values in the bilateral AG indicate that the AG are in a state of hyperactivity during pain signal processing. This may lead to excessive convergence of pain signals within the DMN, impairing the network’s ability to switch to specific pain processing modes. This abnormal neural synchronization may result in amplified perception of pain stimuli and abnormal consolidation of pain-related memories in patients (42). The association between AG dysfunction and heightened pain sensitivity has also been supported by research on migraine; previous studies have found enhanced functional connectivity between the bilateral AG and the left anterior cingulate cortex (ACC) and dorsolateral prefrontal cortex (dlPFC) in migraine patients (43, 44).

As a major DMN node, the PCu is essential for controlling pain sensitivity, processing self-awareness, and integrating resting-state brain activity (45–47). Previous studies have confirmed that it is closely related to pain perception and pain memory formation (48). TTH patients generally have an enhanced response to pain stimuli. The increased NH value of this brain region in this study may reflect its excessive synchronous activation in the resting state, thereby leading to increased pain sensitivity. However, there is a difference from the result of the decreased ReHo value of the left PCu reported by Wang et al. (49). This phenomenon is mainly due to differences in sample size, analysis strategies, and disease course stratification of the research objects. The previous study was an exploratory whole-brain analysis with a small sample of 10 TTH patients, reflecting brain function inhibition in episodic TTH; this study is a large sample involving 68 TTH patients and focuses on specific DMN analysis, which is more consistent with the excessive synchronization of neural activity caused by central sensitization in chronic TTH. At the same time, this echoes the research results of “enhanced PCu functional connectivity and decreased pain threshold” in patients with migraine without aura (6, 50, 51), suggesting that PCu abnormalities may be a common brain network feature of primary headaches (including migraine and TTH), participating in the core regulation process of pain perception.

As a core hub of the DMN, the PCC has core functions, including conscious processing of pain experience, cross-dimensional integration of emotion and pain signals, and regulation of information transmission between brain networks. Its functional abnormalities have been confirmed by a large number of studies to be closely related to the regulatory imbalance of pain regulation pathways and disorders of cognitive-emotional interaction mechanisms (52, 53). In this study, NH analysis found that the NH value of the right PCC in TTH patients was significantly decreased, which suggests that the functional synchronization between this brain region and other nodes within the DMN was significantly impaired, which may further lead to the failure of the descending pain inhibition pathway mediated by it, providing important neuroimaging evidence for the chronic progression of TTH and the formation of central sensitization. This finding is highly consistent with the conclusion of a study on episodic tension-type headache (ETTH) (54): the study used fractional amplitude of low-frequency fluctuations (fALFF) technology and clearly observed that the fALFF value of the PCC region in ETTH patients was significantly decreased, directly reflecting the weakening of the intrinsic neural activity level of this brain region. Although NH and fALFF detect two independent dimensions of “network functional synchronization” and “local neural activity intensity,” both consistently point to the core feature of an abnormal decrease in PCC function, confirming that the functional impairment of this brain region is a common neurobiological manifestation of TTH across disease courses, rather than an isolated phenomenon in specific subtypes or disease stages. At the same time, the results of this study echo the core research conclusions in the previous pain field: whether in acute pain or chronic pain states, the PCC has a clear functional downregulation (53, 55). Structural MRI studies have further provided morphological evidence, confirming that the gray matter volume of the PCC in chronic pain patients is significantly reduced (56), suggesting that functional abnormalities may be accompanied by plastic changes in brain structure; a meta-analysis of functional imaging in the pain field has more clearly verified the specific association between the rostral part of the PCC and pain perception and emotional integration (57). The above multi-dimensional and multi-methodological research evidence mutually confirms that PCC functional abnormality is a key component of the neuropathological mechanism of TTH, and the synergistic effect of its decreased functional synchronization and weakened intrinsic neural activity jointly participates in the disorder of pain regulation and disease progression of TTH.

Notably, we identified a dissociative pattern of NH in the right PCC: patients with TTH exhibited significantly lower PCC NH than healthy controls, yet PCC NH positively correlated with HIT-6, MIDAS scores and headache intensity within the TTH cohort. This divergent pattern implies that PCC NH cannot be regarded as a uniform linear biomarker of headache severity, owing to separate trait- and state-based neuroplastic mechanisms underlying intergroup differences and intra-patient correlations. The group-level reduction in PCC NH reflects trait-like remodeling of the DMN driven by long-standing recurrent headache. Aligned with van Ettinger-Veenstra et al. (58), chronic widespread pain patients display attenuated inferior PCC intrinsic connectivity relative to pain-free individuals; persistent nociceptive input chronically disrupts baseline DMN synchrony, generating a stable disease trait shared across chronic pain populations. Conversely, the positive intra-patient association between PCC NH and clinical metrics arises from state-dependent cross-network modulation. Van Ettinger-Veenstra et al. (58) further reported elevated DMN-salience network (SN) and intra-SN connectivity in patients with heightened pain sensitivity. Ceko et al. (59) systematically verified that patients with more severe pain rely on elevated local PCC synchrony to amplifie self-referential pain rumination and negative affective processing, amplifying subjective headache impairment. This transient PCC hyper-synchronization constitutes a reversible state response scaled to real-time pain intensity, rather than permanent brain reorganization. Therefore, right PCC NH should not be oversimplified as a single linear marker of TTH clinical severity.

Support vector machine technology has been widely used in various biomedical fields. Previous studies have shown that through SVM technology, the ROI-level FC values with significant differences between TTH patients and HCs enabled the SVM classifier to achieve an overall accuracy of 70.476%, sensitivity of 78.790%, specificity of 60.000%, and precision of 68.420%. An ideal diagnostic index should have a sensitivity or specificity of not less than 60%, and a specificity exceeding 70% is helpful for establishing diagnostic criteria (60, 61). The SVM classification results of this study showed that the NH value of the right PCC had an accuracy of 75.57%, a sensitivity of 84.13%, and a specificity of 70.59% in distinguishing TTH patients from healthy controls. Therefore, the NH value of the right PCC can be used as a potential imaging marker to distinguish TTH patients from HCs.

## 6 Limitations

This study had certain limitations: first, the sample size was not further expanded, and no stratified analysis of TTH subtypes (episodic, frequent, and chronic) was performed, making it impossible to clarify the specific differences in NH values of DMN brain regions among patients with different subtypes. Second, this study was a cross-sectional study, which could not reveal the dynamic changes of DMN functional abnormalities in TTH patients or the recovery after intervention. It should be noted that the ROIs in the present correlation analysis were defined based on brain regions with significant intergroup differences within the same dataset. This approach may introduce analytical bias. Therefore, these correlative results should be interpreted cautiously. Further verification using *a priori* ROIs or independent cohorts is warranted. For future studies, the sample size can be expanded to conduct subtype-stratified analysis to clarify the neuroimaging characteristics of TTH with different clinical phenotypes. Meanwhile, longitudinal follow-up studies should be carried out to observe the relationship between NH values of DMN brain regions and the progression of TTH and to explore the improvement effect of neural regulation intervention targeting the right PCC on the clinical symptoms and brain function of patients so as to provide a new direction for the individualized treatment of TTH.

## 7 Conclusion

This study is the first to compare the changes in NH within the DMN between TTH patients and HCs. The results confirmed that TTH patients had functional abnormalities in the core brain regions of the DMN, which were manifested as overactivation of the right SFGmed, bilateral AG, and left PCu, and functional inhibition of the right PCC. Moreover, SVM analysis showed that the NH value of the right PCC exhibited modest discriminative potential to distinguish TTH patients from HCs. Nevertheless, owing to potential circular analysis, this result should be interpreted with caution, and its classification capacity requires further verification in independent cohorts. These findings enrich the research on the neural mechanism of TTH from the perspective of brain network functional abnormalities, provide new imaging evidence for its clinical diagnosis and targeted intervention, and also offer a reference for the research on brain network regulation of chronic pain.

## Data Availability

The original contributions presented in the study are included in the article/supplementary material, further inquiries can be directed to the corresponding author/s.
